# Enhanced receptor binding of SARS-CoV-2 through networks of hydrogen-bonding and hydrophobic interactions

**DOI:** 10.1073/pnas.2008209117

**Published:** 2020-06-05

**Authors:** Yingjie Wang, Meiyi Liu, Jiali Gao

**Affiliations:** ^a^Institute of Systems and Physical Biology, Shenzhen Bay Laboratory, Shenzhen 518055, China;; ^b^College of Chemical Biology and Biotechnology, Beijing University Shenzhen Graduate School, Shenzhen 518055, China;; ^c^Department of Chemistry, University of Minnesota, Minneapolis, MN 55455;; ^d^Minnesota Supercomputing Institute, University of Minnesota, Minneapolis, MN 55455

**Keywords:** protein–protein interaction, SARS-CoV-2, relative free energy of binding, molecular dynamics

## Abstract

Molecular dynamics and free energy simulations have been carried out to elucidate the structural origin of differential protein–protein interactions between the common receptor protein angiotensin converting enzyme 2 (ACE2) and the receptor binding domains of the severe acute respiratory syndrome coronavirus 2 (SARS-CoV-2) [A. E. Gorbalenya *et al.*, *Nat. Microbiol.* 5, 536–544 (2020)] that causes coronavirus disease 2019 (COVID-19) [P. Zhou *et al.*, *Nature* 579, 270–273 (2020)] and the SARS coronavirus in the 2002–2003 (SARS-CoV) [T. Kuiken *et al.*, Lancet 362, 263–270 (2003)] outbreak. Analysis of the dynamic trajectories reveals that the binding interface consists of a primarily hydrophobic region and a delicate hydrogen-bonding network in the 2019 novel coronavirus. A key mutation from a hydrophobic residue in the SARS-CoV sequence to Lys417 in SARS-CoV-2 creates a salt bridge across the central hydrophobic contact region, which along with polar residue mutations results in greater electrostatic complementarity than that of the SARS-CoV complex. Furthermore, both electrostatic effects and enhanced hydrophobic packing due to removal of four out of five proline residues in a short 12-residue loop lead to conformation shift toward a more tilted binding groove in the complex in comparison with the SARS-CoV complex. On the other hand, hydrophobic contacts in the complex of the SARS-CoV–neutralizing antibody 80R are disrupted in the SARS-CoV-2 homology complex model, which is attributed to failure of recognition of SARS-CoV-2 by 80R.

## Significance

Enhanced receptor binding by the severe acute respiratory syndrome coronavirus 2 (SARS-CoV-2) is believed to contribute to the highly contagious transmission rate of coronavirus disease 2019. An understanding of the structural and energetic details responsible for protein–protein interactions between the host receptor ACE2 and SARS-CoV-2 can be useful to epidemic surveillance, diagnosis, and optimization of neutralizing agents. The present study unravels a delicate balance of specific and nonspecific hydrogen-bonding and hydrophobic networks to help elucidate the similarities and differences in receptor binding by SARS-CoV-2 and SARS-CoV.

The novel severe acute respiratory syndrome coronavirus 2 (SARS-CoV-2) that causes the current outbreak of coronavirus disease 2019 (COVID-19) shares many similarities with the SARS coronavirus in 2002–2003 (SARS-CoV), including 76% sequence identity in the spike protein (S) (1–3), a common receptor of the angiotensin converting enzyme 2 (ACE2) (4–6), and the fusion mechanism that involves cleavages of spike at the S1–S2 and S2ʹ sites (7). Amino acid mutations critical to protein–protein interactions have been identified to play a critical role in human-to-human as well as cross-species transmissions (8, 9). Furthermore, it has been reported that the affinity constant for the receptor binding domain (RBD) of SARS-CoV-2 to ACE2 is greater than that of SARS-CoV by as much as a factor of 10 to 15 (10, 11), and the furin recognition sequence “RRAR” at the S1–S2 cleaving site of SARS-CoV-2 represents a near-optimal match for the cellular serine protease TMPRSS2 (5, 12, 13). Both factors likely contribute to the efficiency of virus transmission, making COVID-19 more contagious than infections by SARS-CoV and the influenza virus. Curiously, a SARS-CoV neutralizing antibody, 80R, that recognizes the S protein with nanomolar affinity (14) in the same interfacial region of ACE2 does not show detectable binding to the RBD of SARS-CoV-2 (11, 15). What mutations in the 2019 novel coronavirus make it a stronger binder to ACE2 than SARS-CoV, but, at the same time, capable of evading the antibody against SARS-CoV? An understanding of the underlying mechanisms for protein–protein association between the ACE2 receptor and the RBD of SARS-CoV-2 as well as the difference from that of SARS-CoV is important for virus detection, epidemic surveillance and prevention, and vaccine and inhibitor design.

In this study, we present findings from molecular dynamics (MD) simulations of binary complexes of the RBD domains of both the SARS and COVID-19 viruses with the common receptor ACE2 and the antibody 80R. The present simulations reveal that both electrostatic complementarity and hydrophobic interactions are critical to enhancing receptor binding and escaping antibody recognition by the RBD of SARS-CoV-2.

## Results

### Electrostatic Complementarity Is Enhanced in the RBD–ACE2 Complex of SARS-CoV-2.

**Fig. 1.**
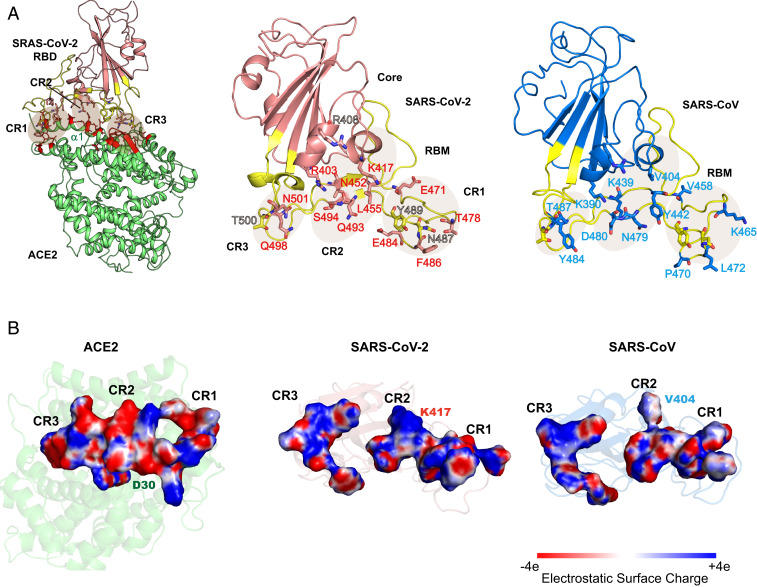
Crystal structures (*A*) and computed electrostatic potentials (*B*) of ACE2 and the RBD. In *A*, the RBD–ACE2 complex is shown (*Left*), along with the designation of binding contact regions CR1, CR2, and CR3 for the RBD of SARS-CoV-2 (*Center*) and SARS-CoV (*Right*); the RBM is colored in yellow, and key residues are shown in stick model. The contact residues in ACE2 is colored in red. In *B*, the van der Waals surface of residues within 3.8 Å between RBD and receptor atoms are colored blue to indicate positive potential and red for negative potential. Crystal structures (PDB ID codes 6LZG and 6ACG) are used for the schematic depiction.

The amino acid sequence of the RBD of SARS-CoV-2 (residue numbers 335 to 515) is highly homologous to that of the SARS-CoV with a single amino acid insertion (Val483) at the edge of the binding interface. Throughout this paper, we use the SARS-CoV-2 sequence number in the discussion and point out the corresponding number for SARS-CoV using a subscript “s.” RBD is structurally divided into a core region, consisting of five antiparallel strands of β-sheet, which is relatively conserved (87.4% sequence identity), and a more variable (50% homology) receptor binding motif (RBM) (*SI Appendix*, Fig. S1). Sequence variations mainly aggregate in the loop regions, two of which are located at both ends of the dimer contact region, denoted as CR1 and CR3 (Fig. 1*A*). Crystal structures show that both RBDs of SARS-CoV-2 and SARS-CoV have the same scaffold (11, 16–18). The middle region in the protein–protein interface (CR2) consists of two short strands of β-sheets bridging across the N-terminal helix of ACE2. While CR3 mainly involves charge-preserving mutations from SARS-CoV to SARS-CoV-2, sequence alterations in the other two regions affect surface electrostatics (Fig. 1*B*). Notably, the V404s→K417 conversion in the 2019 novel coronavirus creates a positive electrostatic patch along with R403 and R408. This change leads to a complementary match with the negative potential from Asp30 over the binding surface of ACE2 (Fig. 1*B*). On the other hand, changes in the CR1 region, including E471(V458_s_), T478(K465_s_), and E484(P470_s_), enhance the negative potential relative to that of SARS-CoV (Fig. 1*B*). Overall, the sequence differences in the RBM enable greater electrostatic complementarity with the receptor ACE2 in SARS-CoV-2 complex than that of SARS-CoV.

### Electrostatic Complementarity Induces Conformational Shift.

**Fig. 2.**
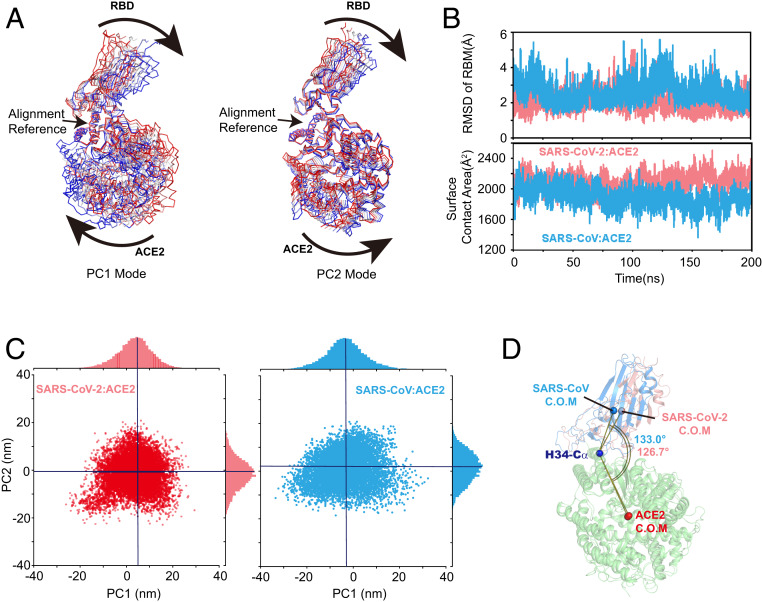
Characteristic dynamic fluctuations of the RBD–ACE2 complexes of SARS-CoV-2 and SARS-CoV depicted by the two lowest-frequency principal components (PC1 and PC2) in *A*, and dynamic conformations projected on to the two principal vectors (*C*). (*B*) The rmsd and contact areas of the receptor-binding motif in both complexes during the 200-ns MD simulations. (*D*) The tilt angles of the two RBD–ACE2 complexes, defined by vectors from the Cα atom of His34 near the center of the N-terminal helix to the centers of mass (C.O.M) for RBD and ACE2, respectively.

Analyses of the trajectories from MD simulations of the binary complexes between ACE2 and the RBDs of both SARS-CoV-2 and SARS-CoV, each lasting 200 ns, reveal that RBD and ACE2 undergo symmetric twist and antisymmetric hinge-bending motions about the axis of the N-terminal helix, corresponding to the lowest quasiharmonic modes, PC1 and PC2, from principal component analysis (Fig. 2*A*). Although the overall conformation dynamics are the same in the two complexes, the average conformation of the SARS-CoV-2 structure is in fact shifted relative to that of SARS-CoV (Fig. 2*C*) when the dynamic configurations are projected onto the two principal vectors. This translates to a net bending of the RBD by about 6° toward the ACE2 binding cleft in the SARS-CoV-2 complex (Fig. 2*D*), while the average configuration of the SARS-CoV is close to the initial crystal structure. Interestingly, comparison of the crystal structures of the two complexes does not show this small but significant conformational difference, perhaps due to crystal packing restraint. Consistent with electrostatic potential complementarity and structural details (discussed below), the observed conformation shift may be attributed to the formation of a salt bridge between Asp30 of ACE2 and Lys417 across the binding groove, along with strengthened loop-anchoring interactions at the bases of the binding interface. The salt bridge is absent in the SARS-CoV complex in which the corresponding Val404_s_ is not in direct contact with ACE2.

Overall, we find that the RBM of SARS-CoV-2 has a relatively smaller root-mean-square deviation (rmsd) in the complex structure than that of SARS-CoV (2.5 vs. 3.0 Å), which is accompanied by a slightly larger surface contact area (Fig. 2*B*), consistent with the suggestion that SARS-CoV-2 has a greater stability than the latter. We have also compared snapshots of structures of the RBD–ACE2 complex of the MD simulations, which are as representative as any other structures of the trajectory, with the four crystal and cryogenic electron microscopy (cryo-EM) structures of SAR2-CoV-2 that have been solved (11, 17, 19, 20). The interfacial interactions explored through MD simulations are in good accord with experiments, with some small but detailed variations such as the uncertainties in side-chain conformation found in different crystal structures (Gln498 and Asn501) and residues in the central region of RBM, including Lys417 and Tyr453 (*SI Appendix*, Fig. S2). The general accord with these structures by cryo-EM and crystallographic methods suggests that the findings from the present MD simulations are consistent with experiments and can be analyzed to gain insights.

### Hydrophobic Contacts Play a Central Role in Anchoring RBD to Its Receptor.

**Fig. 3.**
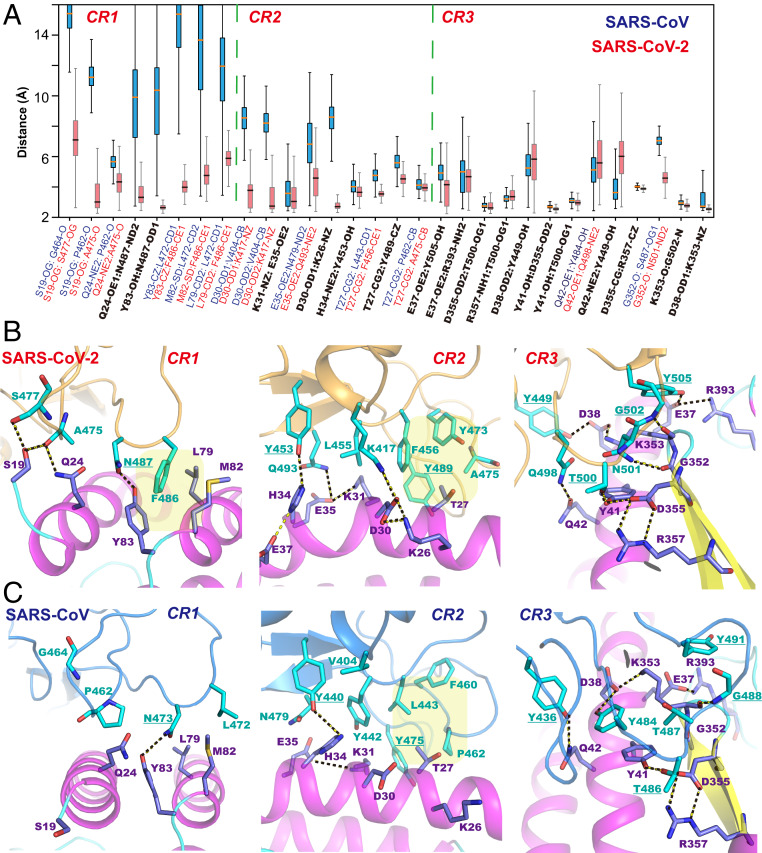
Computed averages and fluctuations of interaction distances of selected residues (*A*) and structural depiction of key interfacial interactions between ACE2 and the RBM of SARS-CoV-2 (*B*) and SARS-CoV (*C*) in the three contact regions at the N-terminal end of ACE2 (CR1), the central region (CR2) of the RBM, and the β-turn contact region of ACE2 (CR3). Key hydrogen bonds and salt bridges are highlighted with dashed lines, and hydrophobic contacts are shaded in yellow background. Legends for *A* are colored light blue for residues in the ACE2–SARS-CoV complex, light maroon for residues in ACE2–SARS-CoV-2, and black for conserved residues found in both sequences at the corresponding sites.

To gain an understanding of the structural origin that governs RBD–ACE2 binding and affinity difference between SARS-CoV-2 and SARS-CoV, we focus on specific amino acid interactions in the three key binding contact regions (Fig. 1*A*). Although many interfacial interactions are duplicated between the two complexes, there are many differences that make SARS-CoV-2 a stronger binder to ACE2 than SARS-CoV (Fig. 3*A*).

The interface between ACE2 and RBD may be roughly divided into hydrophobic and hydrogen-bonding halves. A key feature at the N-terminal end of ACE2 is the hydrophobic contact of Phe486, situated in a pocket fenced by Leu79, Met82, and Tyr83 of ACE2. Tyr83 also donates a hydrogen bond to Asn487 of the RBD, which is preserved in SARS-CoV (Fig. 3*B*). The corresponding hydrophobic residue Leu472_s_ in SARS-CoV is, however, found to point outward, rather than seating in the pocket in the dynamic trajectories, also observed in crystallography (Fig. 3*A* and *C*). Energetically, free-energy perturbation (FEP) simulations for the mutation Leu472_s_Phe resulted in a net change in binding free energy ΔΔG of −1.2 ± 0.2 kcal/mol (Table 1), highlighting the significance of a hydrophobic anchor of the RBM. We attribute the structural difference to increased flexibility in the SARS-CoV-2 sequence due to changes in four out of five Pro residues in a short 12-amino-acid stretch (472 to 483) found in SARS-CoV. It is interesting to note that the Leu472_s_→Phe mutation has been identified previously in a set of five amino acid variations of the original SARS virus, engineered to produce a “superaffinity” binder for ACE2 (21). It is remarkable to see that such an amino acid displacement has naturally occurred in the SARS-CoV-2 sequence.

**Table 1.** Computed relative free energies of binding due to single-site mutations from the receptor ACE2–RBD and the antibody 80R–RBD complexes of SARS-CoV to the corresponding residues in SARS-CoV-2

Mutation	ΔΔG, kcal/mol
SARS-CoV:ACE2	
Y484→Q498	−0.2 ± 0.6
L472→F486	−1.2 ± 0.2
D480→S494, K439→L452	−1.9 ± 0.8
	
T487→N501	−0.5 ± 0.3
N479→Q493	−0.8 ± 0.2
V404→K417, K447→N460*	−2.2 ± 0.9
	
SARS-CoV:80R	
Y484→Q498	−0.2 ± 0.4
L472→F486	−1.4 ± 0.2
D480→S494, K439→L452	3.6 ± 0.5

* Double mutation was performed to keep the system neutral in free energy simulations. Here, K447 is solvent-exposed both in the monomer and in the complex distant from the binding interface and the mutation K447→N460 is expected to cancel out to make minimal contribution to the computed binding free energy.

The interfacial interactions in the central region across the N-terminal helix of ACE2 are dominated by hydrophobic contacts both within the RBM of SARS-CoV-2 itself and across the interface with the receptor. A sequence of hydrophobic contacts aligns over the surface of the N-terminal helix, including Leu455, Phe456, Tyr473, Ala475, and Tyr489, ending with the methyl group of Thr27 of ACE2 tucked in the pocket of the last four residues (Fig. 3*B*). Interestingly, only Tyr489 is retained from the SARS-CoV sequence, whereas the other four hydrophobic residues have been mutated in SARS-CoV-2, respectively, from Tyr442_s_, Leu443_s_, F460_s_, and Pro462_s_ (Fig. 3*C*). Remarkably, although these two sets of hydrophobic residues are quite different in the two RBDs, they form the same type of physical interactions in both complex structures. In view of the structural organization at the interface, it is clear that hydrophobic contacts are preserved and play a significant role to anchor the dimer interface in the RBD–ACE2 complexes.

### Hydrogen-Bonding Network Features Critical Mutations and Specific Interactions.

In contrast to the hydrophobic contacts that dominate RBD and receptor association from the N-terminal (CR1) to the central region (CR2) of the interface, the opposite side of the binding loop (CR3) is characterized by a combination of a delicate network of hydrogen-bonding interactions and a dramatic mutation that produces a salt bridge across the binary interface, distinguishing ACE2 binding of SARS-CoV-2 from that with SARS-CoV. In fact, the most striking difference in the RBD–ACE2 complex between SARS-CoV-2 and SARS-CoV is the Val404_s_-to-Lys417 transition at the apex of the interfacial arch, resulting in an ion pair with Asp30 in the 2019 novel coronavirus. FEP simulations show that the Val→Lys displacement enhances RBD binding to ACE2 by −2.2 ± 0.9 kcal/mol, demonstrating its significant role in ACE2 binding. Another notable variation is Gln493(Asn479_s_), which has been recognized as a key residue whose mutation may be associated with the possible civet (from Arg or Lys)-to-human transmission, presumably due to reduction of electrostatic repulsion with a neighboring “binding hot spot,” Lys31, of ACE2 (22). Extension of the side chain by one carbon (Asn479_s_→Gln493) in SARS-CoV-2 increases free energy of association by −0.8 ± 0.2 kcal/mol (Table 1), although it does not form specific contacts with ACE2 except remote interactions with the Lys31–Asp35 salt bridge on the N-terminal helix.

The binding loop (498 to 505) in CR3 of SARS-CoV-2 (residues 484_s_ to 491_s_ in SARS-CoV) enjoys an extensive hydrogen-bonding network, anchoring the RBD in a groove formed between the turn of an antiparallel β-sheet and the long N-terminal helix of ACE2. At least six amino acids of the RBD and seven residues from ACE2 participate in hydrogen-bonding and ion-pair interactions (Fig. 3*B* and *C*). Asn501 and the backbone of Gly502 each donates a hydrogen bond to the main-chain oxygen atoms of Gly352 and Lys353, respectively. The Gly502–Lys353 pair is preserved in the SARS-CoV complex, but the other hydrogen bond is absent as a result of the amino acid variation of Thr487_s_. We attribute this difference as yet another important factor that enhances the binding association between SARS-CoV-19 and ACE2. This is indeed confirmed by FEP simulations with a computed free energy difference ΔΔG = −0.5 ± 0.3 kcal/mol in the Thr487_s_Asn mutation (Table 1). We note that Thr487_s_ was also selected in the superaffinity RBD of SARS-CoV from a Ser residue (22). It appears that SARS-CoV-2 has found an even stronger variation for receptor binding.

Asp355 on the β-sheet/turn of ACE2 receives a hydrogen bond from Thr500 at the tip of the binding loop. Interestingly, Asp355 itself is involved in an internal (among residues of ACE2) salt bridge with Arg357 as well as a hydrogen bond from Tyr41 of the receptor, which are strictly kept throughout the MD trajectories of all systems investigated in this study. Thr500 is conserved in both SARS coronaviruses, and it is also in close contact with Tyr41 during the dynamic simulations, having an average donor–acceptor distance of 2.7 Å, the same as that with Asp355 (Fig. 3*A*).

Fig. 3*B* shows that the hydrophobic arm of Lys353 is juxtaposed by Tyr41 of ACE2 and Tyr505 of the RBD, extending across the binding groove to form a salt bridge with Asp38 in both complexes. Lys353 has been recognized previously as a (second) receptor binding “hot spot” for SARS-CoV (22), but it does not seem to play a direct role in the RBD–ACE2 complex of SARS-CoV-2. The salt-bridge partner, Asp38, however, forms a transient hydrogen bond with Tyr449 at an average distance of 5.9 Å. Tyr449 is the only residue not in the binding loop of the RBM of SARS-CoV-2 and is preserved in SARS-CoV. The hydrogen-bonding network is completed with the first residue Gln498 of the binding loop, dynamically interacting with Gln42 on the N-terminal helix of ACE2 at an average distance of 6.0 Å. Gln498 replaces the corresponding residue Tyr484_s_ in SARS-CoV, which resulted in only a small perturbation to binding affinity by −0.2 ± 0.6 kcal/mol from free energy calculations. This displacement, however, produces a large effect on the 80R antibody recognition discussed next.

### Disruption of Hydrophobic Contacts Is Likely Responsible for Lack of SARS-CoV-2 Recognition by the SARS-CoV Neutralizing Antibody 80R.

**Fig. 4.**
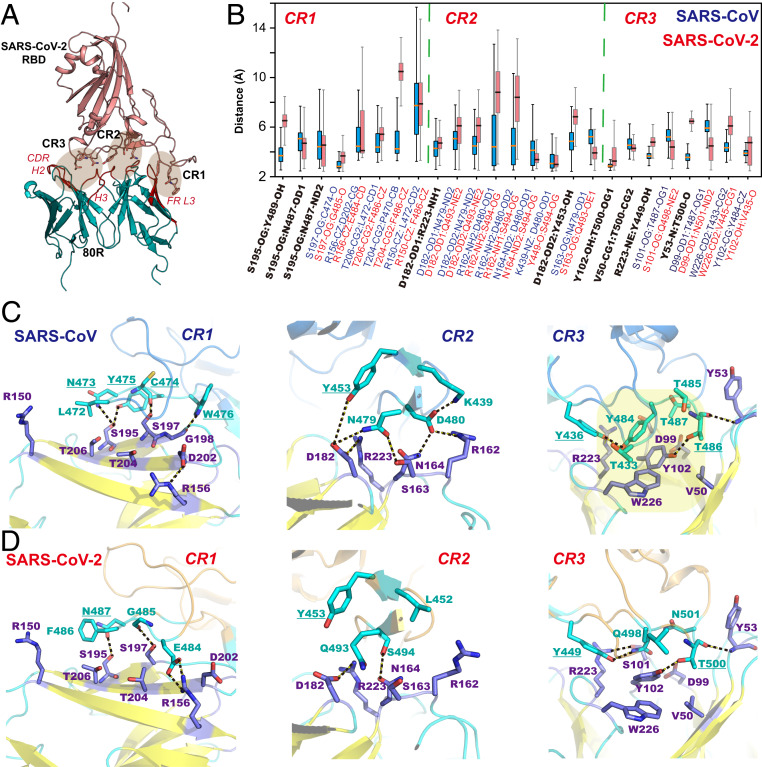
Homology model of the antibody 80R and RBD of SARS-CoV-2 (*A*), computed averages and fluctuations of interatomic distances of selected residues (*B*), and structural details of key interfacial interactions between the SARS-CoV neutralizing antibody 80R and the RBM of SARS-CoV (*C*) and SARS-CoV-2 (*D*) in the three contact regions designated in Fig. 1. In *A*, the CDR loops H2 and H3, mimicking the β-turn binding region of ACE2, and the framework region (FR) loop L3 on 80R are highlighted in red. Key hydrogen bonds and salt bridges are highlighted with dashed lines, and hydrophobic contacts are shaded in yellow background. Legends for *B* are colored light blue for residues in the 80R–SARS-CoV complex, light maroon for residues in 80R–SARS-CoV-2, and black for conserved residues found in both sequences at the corresponding sites.

To this end, we used the crystal structure [Protein Data Bank (PDB) ID code 2GHW (23)] of the 80R–RBD complex of SARS-CoV and built a homology model for its binding to SARS-CoV-2 (Fig. 4*A*) using the former as template to carry out MD simulations for both antibody complexes, each lasting 200 ns, along with FEP calculations. The N-terminal end and central region of the RBD–ACE2 interface is characterized predominantly by hydrophobic contacts, particularly in the SARS-CoV-2 complex (Fig. 3*B* and *C*). In contrast, 80R forms a number of interlocking hydrogen bonds at CR1, including Asn473_s_–Ser195(H), Tyr475_s_–Ser195(H), Cys474_s_–Ser197(H), and Trp476_s_–Gly193(H) (Fig. 4 *B *and *C*), whereas additional hydrophobic contacts can be found with the antibody by Pro469_s_ and Pro470_s_ along with Leu472_s_ on the proline-rich loop. For comparison, many hydrogen bonds are retained [e.g., Asn487–Ser195(H) and Gly485–Ser197(H)] in the homology complex of SARS-CoV-2 (Fig. 4*D*), along with a new ion pair between Glu484 and Arg156(H) thanks to the Pro470_s_→Glu484 mutation. However, it is not clear that this ion pair is stabilizing since it disrupts an internal salt bridge of 80R (Arg156–Asp202), and Asp202 is only 4 Å away from Glu484. Overall, we did not observe obvious structure changes at CR1 to cause 80R losing its affinity for the RBD of SARS-CoV-2. In fact, the Leu472_s_→Phe486 change is predicted to enhance binding by −1.4 ± 0.2 kcal/mol from FEP calculations.

At the opposite end of RBM, CR3 is accommodated by a large hydrophobic pocket composed of both the light and heavy chains of 80R, in sharp contrast to ACE2 binding (Fig. 4*B* and *C*). Notably, the complementarity-determining region (CDR) H2–H3 β-sheet/turn (Fig. 4*C*) mimics an analogous structural element of ACE2 in this location, along with Tyr102 to form a hydrogen bond with Thr486_s_ as that in the RBD–ACE2 complexes. However, this is the only hydrogen bond between 80R and the RBD of SARS-CoV in CR3. Instead, noteworthy is π-stacking between the conserved Tyr484_s_ and Tyr102(L) that constitutes the core of a hydrophobic cluster in the antibody complex (Fig. 4*C*). In SARS-CoV-2, Tyr484_s_ is converted to Gln498, but there is minimal effect on the computed change in binding affinity (−0.2 ± 0.4 kcal/mol). Nevertheless, coupled with amino acid changes of Thr485_s_→Pro499 and Thr487_s_→Asn501 in the binding loop, the hydrophobic core and most of the hydrophobic contacts found in the SARS-CoV complex with 80R are destroyed. The present 200-ns trajectory is too short to see a spontaneous dissociation, but structural disruptions that have been observed suggest that the loss of hydrophobic interactions is a most plausible factor for 80R not to recognize the RBD of SARS-CoV-2.

The structure in the central region of the RBM of SARS-CoV is not very well organized in the 80R complex. The group of hydrophobic residues in contact with the N-terminal helix of ACE2 are rotated and no longer in close proximity to the antibody. An ion pair is found between Asp480_s_ and Arg162, and a hydrogen bond is involved between Asn479_s_ and Asn182 at an average distance of 3.3 Å (Fig. 4*C*). Indeed, single-site mutation of either Asp480_s_Ala or Asp480_s_Gly abolishes binding activity of 80R for the RBD of SARS-CoV (24). However, Asp480_s_ also forms a tight salt bridge with Lys439_s_, which is only 3.6 Å from Arg162. This could counterbalance the stabilizing effect of ion pairing. Thus, double mutation of both Lys439_s_ and Asp480_s_, as in the RBD of SARS-CoV-2, to Leu452 and Ser494, respectively, may not necessarily yield a net destabilizing contribution to binding. Comparison with the ACE2–RBD complexes sheds additional light: Leu452 and Ser494 are not directly in contact with ACE2, and the binding affinity is in fact enhanced by −1.9 ± 0.5 kcal/mol thanks to the double charge annihilation. In the 80R–RBD complex of SARS-CoV, we estimated that these changes reduce binding affinity by 3.6 ± 0.5 kcal/mol, indicating that the salt bridge at Arg162 does play a role in RBD recognition by 80R.

## Discussion

COVID-19 is highly contagious and there is currently no effective agent to combat the infection (25, 26). Its etiological agent, SARS-CoV-2, binds its receptor ACE2 more tightly than SARS-CoV by a factor of 10 to 15, partly contributing to its high infection rate (10). Comparison of crystal and cryo-EM structures and amino acid sequences provided important insights (11, 19, 27); however, it is not clear right away what specific amino acid variations among a large number of changes at the binary interface are responsible for the difference in receptor recognition. A most striking change between the two viruses is the Val404_s_-to-Lys417 mutation, creating an ion pair across the otherwise hydrophobic interface in the central region of the binary complex. Indeed, free energy calculations from our study show that the single-site mutation contributes as much as −2.2 kcal/mol in relative binding affinity, consistent with an overall more compatible electrostatic match between RBD and ACE2 in the complex of SARS-CoV-2 than that in SARS-CoV. Further, in view of the long distance between the two residues, we anticipate that an amino acid mutation from Asp30 to Glu30 in the receptor could also effectively accommodate ion-pair interactions (28).

Analysis of the dynamics trajectories of the binary complexes, however, shows that interfacial interactions are rather complex and it is unlikely that one particular mutation may be singled out as a dominant contributor to the enhanced receptor binding. We found that the cross-section of the binary complex may be roughly divided into a hydrophobic anchor at the N-terminal side and a delicate network of hydrogen bonds on the opposite end of the RBM. Noteworthy is Phe486 in SARS-CoV-2, situated in a hydrophobic pocket of ACE2, whereas the corresponding Leu472_s_ in SARS-CoV points away both in crystal structures and from MD simulation. In SARS-CoV, the CR1 loop is relatively rigid, consisting of five proline residues, four of which are displaced along with the insertion of an extra residue in SARS-CoV-2. Therefore, this loop is more flexible to anchor Phe486 deep into the hydrophobic pocket and more suited for hydrophobic packing along the N-terminal helix than residues in SARS-CoV (e.g., Tyr473 points toward ACE2, but Phe460_s_ in SARS-CoV faces an orthogonal direction). A long patch of hydrophobic residues is found over the N-terminal helix of ACE2, extending to the middle region of the RBM in both complexes, but the amino acids involved are all different except one. Interestingly, there are no specific side-chain contacts from the receptor ACE2, except the methyl group of Thr27. These observations suggest that nonspecific, hydrophobic aggregation is key to initiate contact between ACE2 and RBD. Thus, it is expected that mutations of these residues, as long as they are hydrophobic in nature, would not make a large impact on binding, but together they play a critical role in complex formation.

The other end of the RBM involves a series of finely connected hydrogen-bonding interactions in the SARS-CoV-2 complex. A noticeable difference from that of SARS-CoV is the Thr487_s_-to-Asn501 transition; MD simulations show that Asn501 makes a second hydrogen bond to the main-chain oxygen of Gly352 of ACE2, contributing about −0.5 kcal/mol in binding free energy [the other connection between main chains of Gly502 and Lys353(ACE2) is conserved in both complexes]. The Thr487_s_→Asn501 mutation also affects hydrophobic stacking of Tyr41(ACE2)–Lys353(ACE2)–Tyr505 to become more ordered in SARS-CoV-2 than in SARS-CoV, favoring hydrogen-bonding interactions involving these residues. Contrary to a previous suggestion as a binding hot spot in the SARS-CoV recognition (22), there seems to be no specific role for Lys353 in binding other than stabilizing the internal configuration of ACE2 by forming an ion pair with Asp38. Consequently, a mutation either in RBD or ACE2 that stabilizes the hydrophobic stacking could be favorable for binding.

Following the 2003 SARS epidemic, many neutralizing antibodies have been isolated and a number of crystal structures are available (14, 29, 30), among which 80R is particularly interesting because it binds the RBD of SARS-CoV in an orientation similar to the native receptor (23). Yet, 80R showed no activity against SARS-CoV-2 (15), in contrast to a different antibody, CR3022, that binds both SARS-CoV and SARS-CoV-2 at an orthogonal binding site (31). What makes an antibody that binds its antigen in a way similar to the native receptor, but is incapable of recognizing a closely related target that shares the same receptor? An understanding of this question would be useful for designing a neutralizing agent for SARS-CoV-2 recognition.

At first glance, 80R recognizes the RBD of SARS-CoV in a fashion eerily similar to ACE2, making numerous contacts with a similar set of residues (Fig. 4*B* and *SI Appendix*, Tables S1 and S2). For example, the CDR of the H2–H3 β-sheet/turn is analogous to the same structural element of ACE2 in this location, and the hydrogen bond between Tyr102(H) and Thr486_s_ is identical to that in the RBD–ACE2 complexes. Nevertheless, the specific details at the contact regions are different. The hydrophobic and hydrogen-bonding regions of the RBM of SARS-CoV are reversed in the antibody 80R complex in comparison with the ACE2 complex. Importantly, the ion pair between Asp480_s_ and Arg162 in the SARS-CoV complex is not feasible in SARS-CoV-2 because of the Ser494 mutation, but an internal salt bridge with Arg439_s_ is only 3.3 Å from Arg162(L), making it unclear whether or not the net effect of this salt bridge is a stabilizing contribution. Free energy calculations show that double mutation of the internal ion pair of SARS-CoV to Leu452 and Ser494, the corresponding residues in SARS-CoV-2, reduces binding free energy by 3.6 kcal/mol, sufficient to account for the loss of activity for 80R to recognize SARS-CoV-2. However, in the ACE2–RBD complex, the same double mutation in fact stabilizes the SARS-CoV-2 complex by −1.9 kcal/mol. Finally, we note that the CR3 region is hosted by a large hydrophobic pocket with a core π-stacking between Tyr484_s_ and Tyr102(H) of the antibody, surrounded by a cluster of hydrophobic contacts. In SARS-CoV-2, Tyr484_s_ is replaced by Gln498, and along with other mutations the hydrophobic interactions are disrupted in this region. Thus, disruption of hydrophobic contacts with 80R in the CR3 region of SARS-CoV-2 is critically responsible for a lack of detectable binding.

Previous structural analyses and mutagenesis studies suggest that several residues changing from SARS-CoV to SARS-CoV-2 may enhance binding affinity (17, 20, 32). Our simulation results help clearly identify the interplay of differential hydrophobic contacts on one side of the RBM and electrostatic complementarity and hydrogen-bonding network extended to the opposite end (27). On the surface, the overall binding mode of the neutralizing antibody 80R for the RBD of SARS-CoV is similar to that of ACE2, but the hydrophobic and hydrogen-bonding sites are reversed. Modeling of a homology complex indicates that key amino acid displacements in the 2019 novel coronavirus disrupt hydrophobic contacts and along with annihilation of an ion pair are responsible for 80R’s not recognizing SARS-CoV-2. Future studies aimed at an understanding of the specific roles of posttranslation modification in protein–protein interactions would be important (31, 33–35).

## Materials and Methods

### MD Simulation.

The crystal structures of binary complexes between ACE2 and the RBD (PDB ID codes 6ACG and 6LZG) were used to initiate the MD simulations (11, 18). The protonation states of histidine residues were determined on the basis of local hydrogen-bonding interactions. The protein was placed in a dodecahedron unit cell of water molecules represented by the three-point charge TIP3P model (36), whose boundary is at least 11 Å from any protein atoms. The solvated protein was subsequently neutralized and filled with a concentration of 0.13 M of KCl salt. Covalent bonds involving hydrogen atoms were constrained using the LINCS algorithm (37), and long-range electrostatic interactions were treated with particle-mesh Ewald employing a real-space cutoff of 10 Å (38). The system was first briefly minimized with backbone atoms restrained to the crystal coordinates to remove close contacts, and the restrained system was gradually heated to 300 K under constant volume conditions in 1 ns. The harmonic restraints were gradually released following the next 5 ns of simulations using the constant isothermal–isobaric ensemble at 1 atm and 300 K. Each system was equilibrated for at least an additional 10 ns, without any restraints. The Parrinello–Rahman (39) barostat and a V-rescale thermostat were used with an integration time step of 2 fs. MD simulations were extended for 200 ns with coordinates recorded every 20 ps. All simulations were performed using GROMACS 5.1.4 (40) along with the CHARMM36 force field (41).

The same procedure was followed for simulations of the antibody 80R–ACE2 complex using a crystal structure for the RBD of SARS-CoV (PDB ID code 2GHW) (23) and a homology model for SARS-CoV2 generated using Swiss-model Server (42) based on the SARS-CoV structure.

### Relative Free Energy of Binding.

The relative free energies of binding ΔΔG due to amino acid mutations were determined following a protocol based on the Bennet acceptance ratio implemented in GROMACS 5.1.4 (43). The procedure employs dual protein topologies that include both residues of the wild-type (λ = 0) and the mutant protein (λ = 1) coupled by the progressing variable λ. Of course, both the complex and unbound structures were used to obtain the change in binding free energies using a standard thermodynamic cycle approach. Single-site mutations were performed based on the SARS-CoV structure except for a few cases noted in the text. The computational details are identical to those detailed above, except that after 20 ns of equilibration of both initial and final states for each mutation 100 additional trajectories, each lasing 100 ps, were initiated both in the forward and in the backward transformations to accumulate statistical averages and fluctuations.

### Data Availability.

The initial crystal structures for the protein complexes are taken from the PDB (https://www.rcsb.org) with the PDB ID codes indicated in the text. The homology model complex between antibody 80R and SARS-CoV-2 is provided as *SI Appendix*, along with data used to generate Figs. 2–4 from simulations carried with the GROMACS program, which is freely available at http://www.gromacs.org under the GNU Lesser General Public License.

## Supplementary Material

Supplementary File

Supplementary File

Supplementary File
