# Supplementary material for: Enhanced receptor binding of SARS-CoV-2 through networks of hydrogen-bonding and hydrophobic interactions
Source: Proc Natl Acad Sci U S A. 2020 Jun 5;117(25):13967–74. doi: 10.1073/pnas.2008209117 (PMC7322019; doi:10.1073/pnas.2008209117)
Supplement: Supplementary File [file pnas.2008209117.sapp.pdf]

PNAS
www.pnas.org

Supplementary Information for

Enhanced Receptor Binding of SARS-CoV-2 through Networks of Hydrogen-bonding and Hydrophobic Interactions

Yingjie Wang,[1] Meiyi Liu,[1,2] and Jiali Gao[1,2,3]

Jiali Gao
Email: gao@jialigao.org

This PDF file includes:

Figures S1 to S2
Tables S1 to S4

www.pnas.org/cgi/doi/10.1073/pnas.2008209117

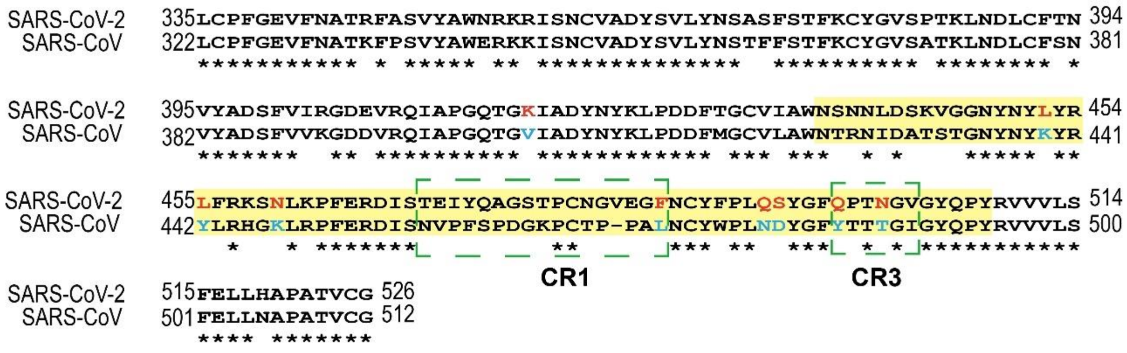

Figure S1. Comparison of sequence of receptor binding domain (RBD) between SARS-CoV-2 and SARS-CoV, highlighting CR1 and CR3 in RBM that is colored in yellow, and the key variation is colored in red for SARS-CoV-2, and blue for SARS-CoV.

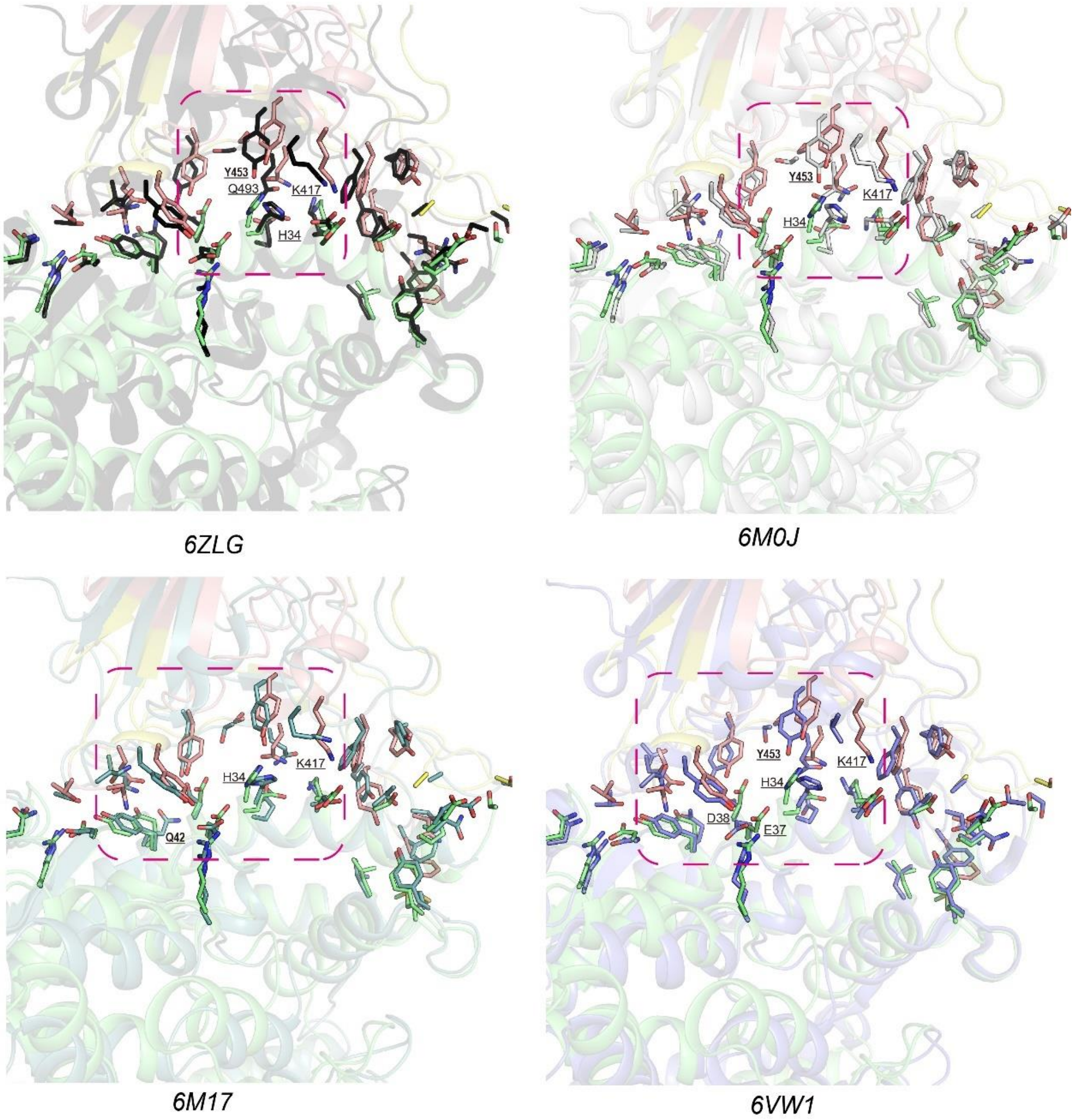

Figure S2. Comparison of the MD snapshot of SARS-CoV-2 RBD: ACE2 complex with the available experimental structures that are denoted by PDB ID. Overall, the protein-protein interface is highly similar, with slight differences in CR2 highlighted in squares.

Table S1. Contact surface residues in SARS-CoV:ACE2 and SARS-CoV-2:ACE2 complexes.

SARS-CoV: ACE2		SARS-CoV-2:ACE2	
SARS-CoV Residue	***ACE2 Residue***	***SARS-CoV-2 Residue***	***ACE2 Residue***
Y436	S19	Y449	S19
Y440	Q24	Y453	Q24
Y442	T27	L455	K26
L443	K31	F456	T27
P462	H34	Y473	D30
F469	E35	A475	K31
L472	E37	S477	H34
N473	D38	F486	E35
Y475	Y41	N487	E37
Y484	Q42	Y489	D38
T486	L79	Q493	Y41
T487	M82	Q498	Q42
G488	Y83	T500	L79
Y491	G352	N501	M82
	K353	G502	Y83
	D355	Y505	G352
	R357		K353
	R393		D355
			R357
			R393

Table S2. Contact surface residues in SARS-CoV:80R and SARS-CoV-2:80R complexes.

SARS-CoV: 80R		SARS-CoV-2:80R	
SARS-CoV Residue	***80R Residue***	***SARS-CoV-2 Residue***	***80R Residue***
T433	V50	Y449	D99
Y436	Y53	E484	S101
K439	D99	G485	Y102
Y453	S101	F486	R156
L472	Y102	N487	S163
N473	R156	Q493	N164
C474	R162	S494	D182
Y475	S163	Q498	S195
W476	N164	T500	S197
N479	D182	N501	D202
D480	S195		T204
Y484	S197		T206
T485	G198		R223
T486	D202		
T487	T204		
	T206		
	R223		
	W226		

Table S3. Statistics of interfacial hydrogen bond (H-bond) in SARS-CoV:ACE2 and SARS-CoV-2:ACE2 complexes.

	SARS-CoV: ACE2		SARS-CoV-2:ACE2	
	H-bond Pair	***d(Å)***	***H-bond Pair***	***d(Å)***
CR1	Q24-NE2:P462-O	5.5±1.0	S19-OG:S477-OG	7.4±1.7
			S19-OG:A475-O	3.8±1.4
			Q24-NE2:A475-O	4.4±0.9
			Q24-OD1: N487-ND2	3.9±1.1
			Y83-OH:N487-OD1	2.9±0.4
CR2	H34-NE2:Y441-OH	4.1±0.6	D30-OD1:K417-NE	3.7±1.0
	K31-NZ:E35-OE2	3.7±1.1	D30-OD2:K417-NE	3.4±0.9
			H34-NE2:Y454-OH	3.8±0.5
			K26-NZ:D30-OD1	3.4±1.5
			E35-OE2:N493-NE2	4.6±1.2
			K31-NZ:E35-OE2	3.6±1.1
CR3	E37-OE2:Y491-OH	4.9±0.9	E37-OE2:Y505-OH	4.0±1.0
	E37-OE2:R393-NH2	4.8±1.2	E37-OE2:R393-NH2	4.6±0.9
	D355-OD2:T486-OG1	3.0±0.6	D355-OD2:T500-OG1	3.1±0.9
	R357-NH1:T486-OG1	3.3±0.5	R357-NH1:T500-OG1	3.7±0.8
	D38-OD2:Y436-OH	5.5±1.2	D38-OD2:Y449-OH	5.9±2.4
	Y41-OH:D355-OD2	2.7±0.2	Y41-OH:D355-OD2	2.7±0.2
	Y41-OH:T486-OG1	3.1±0.3	Y41-OH:T500-OG1	3.1±0.4
	Q42-OE1:Y484-OH	5.1±1.2	Q42-OE1:Q498-NE2	6.0±1.6
	Q42-NE2:Y436-OH	4.0±1.2	Q42-NE2:Y449-OH	6.3±2.2
	R355-CG:R357-CZ	4.0±0.1	R355-CG:R357-CZ	4.0±0.1
	K353-O:G488-N	3.0±0.3	G352-O:N501-ND2	4.8±0.6
	D38-OD1:K353-NZ	3.2±0.8	K353-O:G502-N	2.9±0.2
			D38-OD1:K353-NZ	2.9±0.6

Table S4. Statistics of interfacial hydrogen bond (H-bond) in SARS-CoV:80R and SARS-CoV-2:80R complexes.

	SARS-CoV: 80R		SARS-CoV-2:80R	
	H-bond Pair	***d(Å)***	***H-bond Pair***	***d(Å)***
CR1	S195-OG:Y475-OH	3.9±0.9	S195-OG:N487-OD1	4.5±1.2
	S195-OG:N473-OD1	4.8±1.1	S195-OG:N487-ND2	4.3±1.3
	S195-OG:N473-ND2	4.7±1.4	S197-OG:G485-O	3.7±0.8
	S197-OG:C474-O	3.3±1.1	R156-CZ:E484-CD	5.9±2.8
	R156-CZ:D202-CG	5.2±1.7		
CR2	D182-OD1:R223-NH1	4.3±0.9	D182-OD1:R223-NH1	4.7±0.7
	D182-OD1:N479-ND2	4.9±1.1	D182-OD1:Q493-NE2	5.8±1.2
	D182-OD2:N479-ND2	4.6±1.0	D182-OD2:Q493-NE2	5.8±1.3
	R162-NH2:D480-OD1	5.1±2.5	N164-ND2:S494-OG	3.6±0.7
	R162-NH2:D480-OD2	5.1±2.4	Y449-O:S494-OG	3.3±0.6
	N164-ND2:D480-OD1	3.9±1.0	S163-OG:Q493-OE1	4.0±0.8
	K439-NZ:D480-OD1	3.3±0.8		
	D182-OD2:Y440-OH	4.9±1.0		
	S163-OG:N479-OD1	5.2±0.9		
CR3	Y102-OH:T486-OG1	3.0±0.6	Y102-OH:T500-OG1	3.7±1.0
	R223-NE:Y449-OH	3.9±0.7	R223-NE:Y449-OH	4.8±0.5
	S101-OG:T487-OG1	5.4±0.8	S101-OG:Q498-NE2	4.3±0.9
	D99-OD1:T487-OG1	6.1±0.6	D99-OD1:N501-ND2	4.4±1.2
	Y53-N:T486-O	3.7±0.7	Y102-OH:V435-O	4.3±1.1
